# An intronic *PICALM* polymorphism, rs588076, is associated with allelic expression of a *PICALM* isoform

**DOI:** 10.1186/1750-1326-9-32

**Published:** 2014-08-29

**Authors:** Ishita Parikh, Christopher Medway, Steven Younkin, David W Fardo, Steven Estus

**Affiliations:** 1 Departments of Physiology, Sanders-Brown Center on Aging, University of Kentucky, 800 S. Limestone St., Lexington, KY 40536, USA; 2 Department of Neuroscience, Mayo Clinic Jacksonville, Jacksonville, FL 32224, USA; 3 Biostatistics, Sanders-Brown Center on Aging, University of Kentucky, 800 S. Limestone St., Lexington, KY 40536, USA

**Keywords:** *PICALM*, Alzheimer’s disease, Next-generation sequencing, Allelic expression imbalance, Single nucleotide polymorphism

## Abstract

**Background:**

Although genome wide studies have associated single nucleotide polymorphisms (SNP)s near *PICALM* with Alzheimer’s disease (AD), the mechanism underlying this association is unclear. PICALM is involved in clathrin-mediated endocytosis and modulates Aß clearance *in vitro*. Comparing allelic expression provides the means to detect cis-acting regulatory polymorphisms. Thus, we evaluated whether *PICALM* showed allele expression imbalance (AEI) and whether this imbalance was associated with the AD-associated polymorphism, rs3851179.

**Results:**

We measured *PICALM* allelic expression in 42 human brain samples by using next-generation sequencing. Overall, *PICALM* demonstrated equal allelic expression with no detectable influence by rs3851179. A single sample demonstrated robust global *PICALM* allelic expression imbalance (AEI), i.e., each of the measured isoforms showed AEI. Moreover, the *PICALM* isoform lacking exons 18 and 19 (*D18-19 PICALM*) showed significant AEI in a subset of individuals. Sequencing these individuals and subsequent genotyping revealed that rs588076, located in *PICALM* intron 17, was robustly associated with this imbalance in *D18-19 PICALM* allelic expression (p = 9.54 x 10^-5^). This polymorphism has been associated previously with systolic blood pressure response to calcium channel blocking agents. To evaluate whether this polymorphism was associated with AD, we genotyped 3269 individuals and found that rs588076 was modestly associated with AD. However, when both the primary AD SNP rs3851179 was added to the logistic regression model, only rs3851179 was significantly associated with AD.

**Conclusions:**

*PICALM* expression shows no evidence of AEI associated with rs3851179. Robust global AEI was detected in one sample, suggesting the existence of a rare SNP that strongly modulates *PICALM* expression. AEI was detected for the *D18-19 PICALM* isoform, and rs588076 was associated with this AEI pattern. Conditional on rs3851179, rs588076 was not associated with AD risk, suggesting that *D18-19 PICALM* is not critical in AD. In summary, this analysis of *PICALM* allelic expression provides novel insights into the genetics of *PICALM* expression and AD risk.

## Background

Phosphatidylinositol binding clathrin assembly protein (*PICALM*) facilitates clathrin-mediated endocytosis. PICALM binds phosphatidylinositol 4,5- bisphosphate (PIP_2_), adaptor protein 2 (AP_2_) and clathrin to mediate endocytic clathrin coated vesicle formation at the plasma membrane. Although PICALM is ubiquitously expressed, PICALM expression is more pronounced in microvessels [1,2]. Previous studies have shown PICALM co-localizes with APP and modulates amyloid beta (Aß) generation [3-5]. Accumulation of Aß deposits is a hallmark of Alzheimer’s Disease (AD) pathology.

Genome wide association studies in multiple cohorts have identified single nucleotide polymorphisms (SNP)s near the *PICALM* gene as significantly associated with AD risk [6-10]. Studies were first conducted with Caucasian populations and then independently verified in several although not all Asian populations [11-15]. These studies report that the rs3851179 A allele reduces AD risk with an odds ratio of 0.88 [6]. This SNP is located approximately 80 kb 5′ of *PICALM.*

Understanding how rs3851179 alters *PICALM* to impact AD risk may lead to novel insights into AD mechanisms and potential treatments. Since rs3851179 is not in linkage disequilibrium (LD) with a SNP that alters a *PICALM* amino acid (r^2^ < 0.1), we hypothesize that rs3851179 is associated with changes in mRNA transcription or processing. Allelic expression imbalance (AEI), which is an expression difference between allelic transcripts within an individual, has been used to detect *cis*-regulatory effects [16-20]. Here, we performed an AEI analysis by comparing allelic expression through the use of two exonic SNPs, rs76719109 and rs592297, in AD and non-AD brain samples. These studies included 35 samples that were heterozygous for rs76719109 and 19 samples that were heterozygous for rs592297. While *PICALM* expression did not show AEI overall, one individual showed robust *PICALM* AEI, with an allelic ratio of 0.76. Additionally, significant AEI was detected for the *PICALM* isoform lacking exons 18 and 19 (*D18-19 PICALM*). Sequencing and additional genotyping established that rs588076 was robustly associated with this AEI pattern. Interestingly, rs588076 has been associated with blood pressure response to ^Ca++^ channel blocking agents [21]. We discuss these overall results in the context that genetic regulation of *PICALM* isoforms relative to AD risk is highly complex with further work necessary to elucidate the mechanisms modulating genetic risk.

## Results

**Figure 1:**
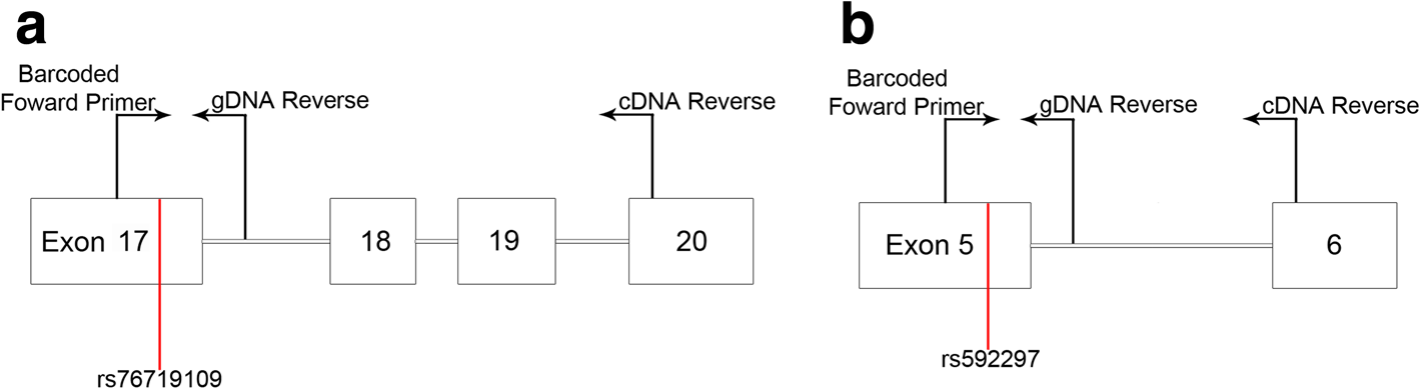
**Rs76719109 and rs592297 AEI assays. a)** For the exon 17 SNP rs76719109, a barcoded forward primer was positioned in exon 17, and a reverse primer was positioned in intron 17 (genomic samples) or exon 20 (cDNA samples). **b)** For the exon 5 SNP rs592297 assay, a barcoded forward primer was positioned in exon 5, and a reverse primer was positioned in intron 5 (genomic samples) or exon 6 (cDNA samples).

**Figure 2:**
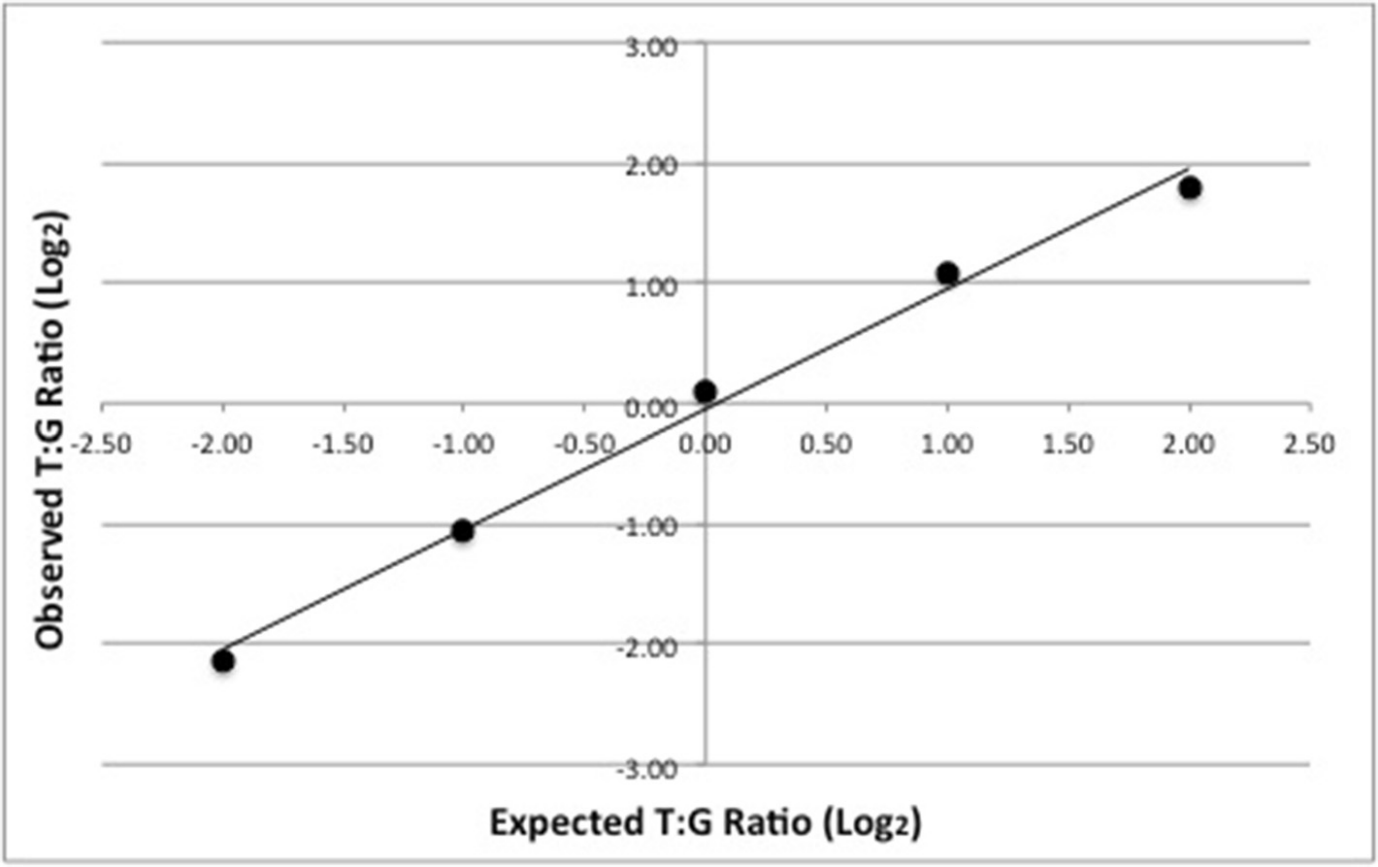
**Linearity of allelic expression assay.** Different proportions of rs76719109 T and G homozygous cDNA were mixed to test the linearity of the AEI assay. The T:G ratios were 1:4, 1:2, 1:1, 2:1, and 4:1. An overall linear relationship was found (r^2^ = 0.99). The slope was 0.999, i.e., the assay detected the T and G alleles with equal efficiency. The graphs are plotted log_2_ to avoid compression at the lower ratios and thereby better visualize the data [22].

**Figure 3:**
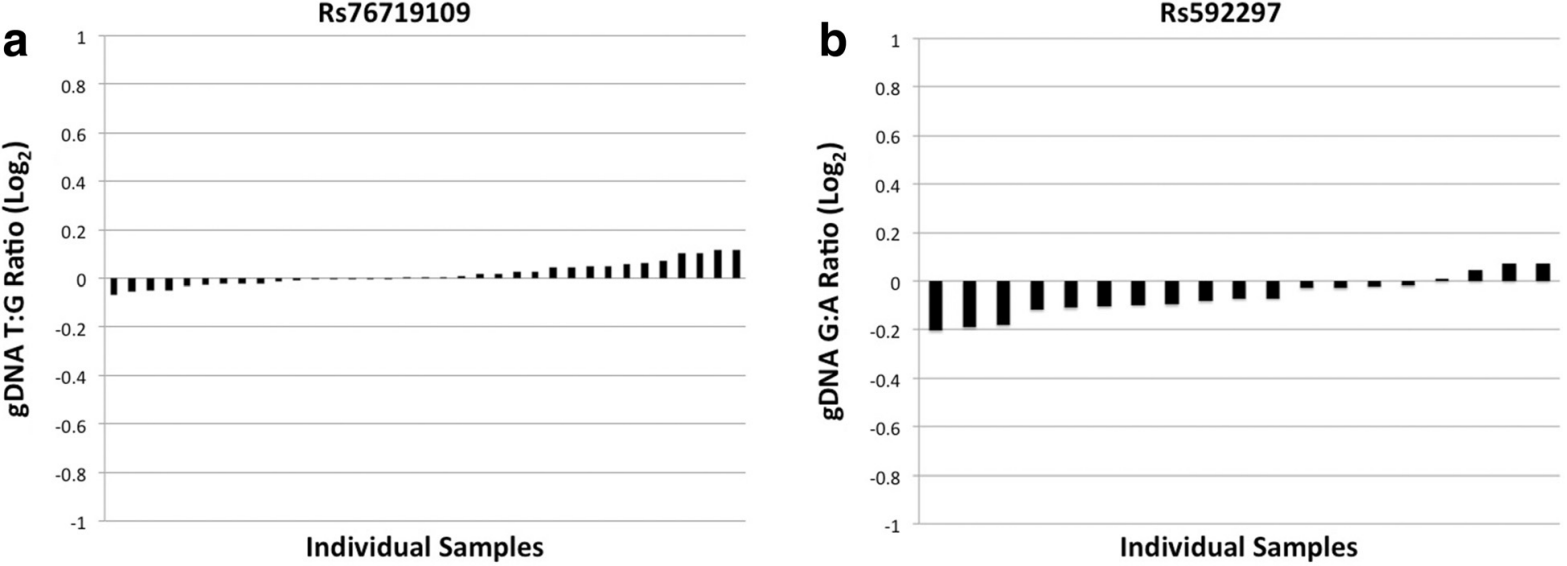
**Genomic DNA Allelic Ratios. a-b)** The allelic ratio for the gDNA samples for rs76719109 and rs592297 is shown. None of the samples showed significant AEI with each of the samples consistently near the expected 1:1 ratio (note that a 1.0 ratio is equal to 0 in log_2_).

To detect the presence of regulatory cis-acting SNPs in human brain samples, we measured allelic ratios in cDNA from reverse transcribed mRNA. Heterozygosity for an exonic "reporter" SNP provides the means to compare the expression of one allele with another allele within an individual. Our criteria for reporter SNPs for AEI analysis is that the SNPs are present in exons and have a minor allele frequency (MAF) greater than 15%, which allows for sufficient sample numbers for analysis. Only two PICALM SNPs satisfied these criteria, rs76719109 and rs592297 (Figure 1). Rs76719109 has a MAF of 0.44 and resides within exon 17; PCR amplification from exon 17–20 allowed us to measure AEI for total *PICALM* as well as *PICALM* splice variants lacking exon 18 or exons 18–19 (Figure 1a). Rs592297 has a MAF of 0.20 and resides in exon 5. PCR amplification from exon 5–6 produced a single PCR product for cDNA (Figure 1b). The AEI assay was validated in two ways. First, we tested the linearity of the assay by generating a cDNA standard curve consisting of five different rs76719109 T:G ratios (Figure 2). Our input T:G ratios ranged from 1:4 to 4:1. We found a robust linear relationship between input and observed T:G ratios. Second, we applied our experimental approach to genomic DNA (gDNA), which represented a positive control with an expected "allelic" ratio of 1.0. Rs76719109 and rs592297 showed gDNA ratios of 1.01 ± 0.03 (mean ± SD, n = 35) and 0.96 ± 0.05 (mean ± SD, n = 19), respectively (Figure 3). Hence, this AEI assay appears robust for detecting and quantifying variations in allelic expression.

**Figure 4:**
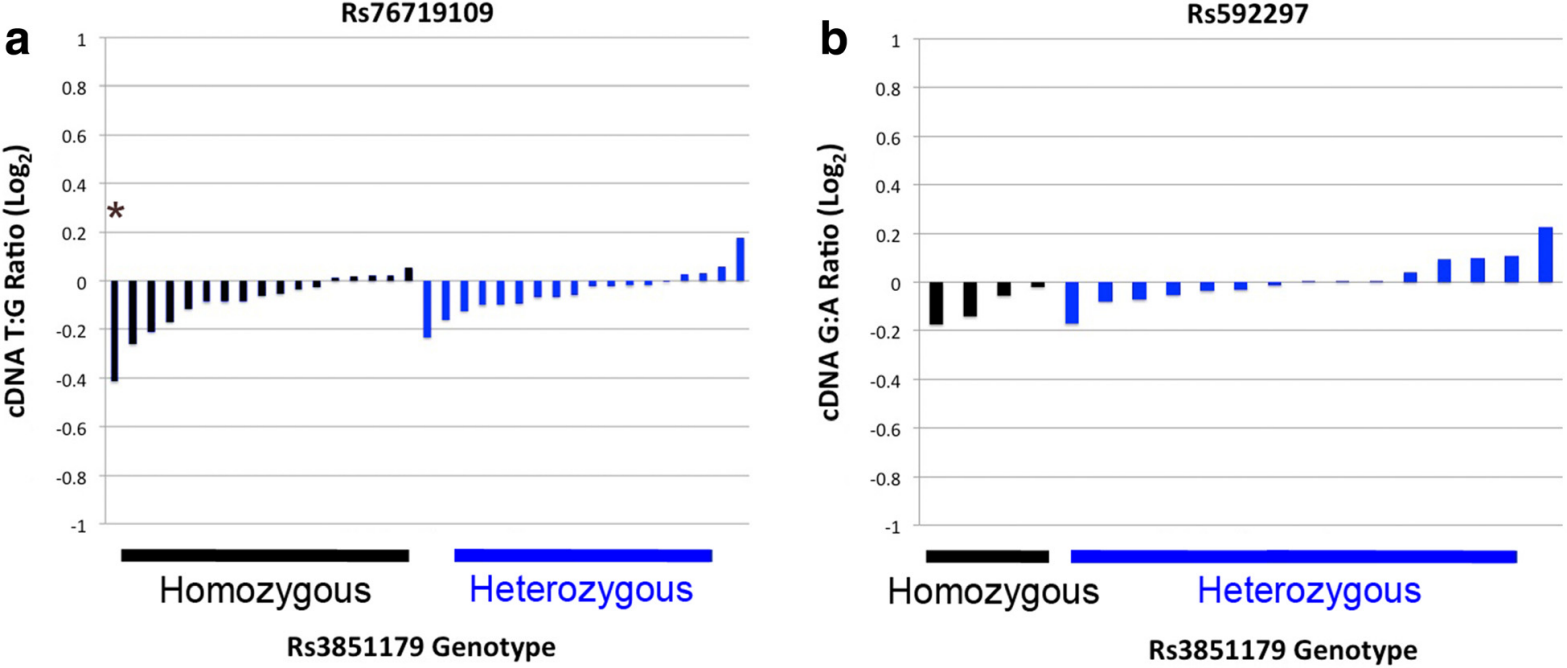
**Evaluation of total *PICALM *AEI with respect to rs3851179. a-b)** Allelic *PICALM* expression was assessed by rs76719109 or rs592297. Each individual sample was normalized to its gDNA ratio. Rs3861179 was not associated with significant AEI, i.e., only one sample (*) showed significant AEI.

To evaluate whether the AD-associated SNP rs3851179 was associated with unequal allelic *PICALM* expression, we performed AEI analysis with rs76719109 and rs592297on a total of 54 samples. Twelve of these 54 samples were heterozygous for both SNPs. Hence, we analyzed *PICALM* for AEI in a total of 42 unique individual samples. This effort analyzed 4.2 million sequences for rs76719109 and 1.4 million sequences for rs592297. If rs3851179 modulated total *PICALM* expression, we expected to see significant AEI in individuals heterozygous for rs3851179, but not in individuals homozygous for rs3851179. When we analyzed the results for the exon 17 SNP, rs76719109, significant AEI was observed in only a single sample, termed AD40 (see below). To evaluate whether a subtle difference in allelic expression may be present and associated with rs3851179, we compared the mean allelic expression between the rs3851179 homozygous and heterozygous groups. This approach did not discern a significant difference between the two groups, i.e., the allelic ratios for rs3851179 homozygous and heterozygous groups was 0.94 ± 0.08 (n = 17) and 0.97 ± 0.06 (n = 18), respectively (Figure 4a, p = 0.24, t-test). Hence, rs3851179 did not appear associated with *PICALM* AEI. To confirm this finding and extend the analysis to additional samples, we analyzed allelic expression by using the exon 5 SNP, rs592297. Significant AEI was not observed in any sample, noting that the sample with the significant AEI result from the rs76719109 analysis was homozygous for rs592297 and not suitable for evaluation. When the allelic ratios were analyzed by t-test to evaluate whether rs3851179 was associated with allelic expression, the findings confirmed that rs3851179 was not associated with robust AEI; the allelic ratios were 0.93 ± 0.05 (n = 4) for rs3851179 homozygotes and 1.01 ± 0.07 (n = 15) for heterozygotes (Figure 4b, p = 0.06). Hence, we found that the total *PICALM* allelic ratio for each person was remarkably consistent with both reporter SNPs; rs3851179 is not associated with overall allelic *PICALM* expression.

Interestingly, cDNA from the individual termed AD40 showed significant unequal allelic mRNA expression with a T:G ratio of 0.76 (Figure 4a, Additional file 1: Table S1). This ratio is based on a total of four experiments that detected a total of 68141 copies of the T allele and 88400 copies of the G allele. This AEI was not due to genomic normalization as the genomic T:G ratio was 1.01. These data are based upon the rs76719109 SNP because this individual was homozygous for rs592297. A similar result was obtained when each of four separate replicates were analyzed individually, i.e., when each replicate was analyzed individually, the T:G ratio was 0.73 ± 0.04 (mean ± SD). Hence, significant AEI was observed in a single individual among the 42 unique samples.

**Figure 5:**
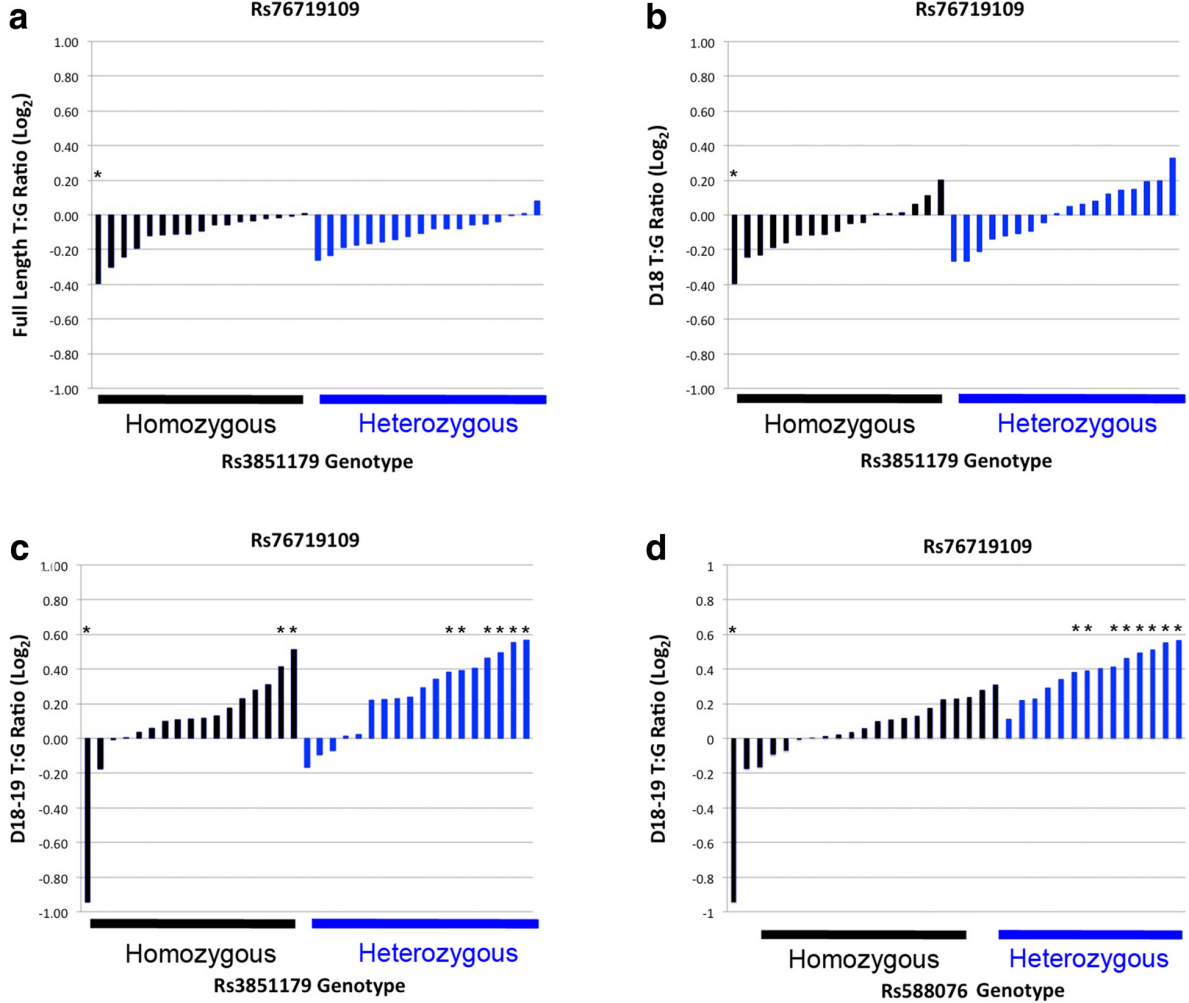
**Evaluation of *PICALM *isoform AEI with respect to rs3851179.** The indicated *PICALM* isoforms were analyzed for AEI as a function of rs3851179 **(a-c)** or rs588076 **(d)**. Each allelic ratio was normalized to the sample’s gDNA ratio. **a)** The *full length PICALM* isoform contained exons 18–19 and showed equal allelic ratios, with a non-significant trend towards an increase in the G allele. **b)***D18-PICALM* showed equal allelic ratios and **c)***D18-19 PICALM* showed significant unequal allelic ratios in 9 samples (*p < 0.05), **d)** The *D18-19 PICALM* AEI was associated with rs588076 heterozygosity. This pattern of significant AEI was not associated with sex, age or AD status (p > 0.05).

Having considered overall *PICALM* allelic expression, we proceeded to apply this AEI analysis to *PICALM* isoforms. The exon 17 to exon 20 PCR amplicons captured three different *PICALM* isoforms because exons 18 and 19 are variably spliced. These isoforms were termed *full length PICALM* (contains exons 18–19), *D18*-*PICALM* (lacked exon 18) or *D18-19 PICALM* (lacked both exons 18 and 19). We analyzed each of these isoforms for the presence of AEI as a function of rs3851179 heterozygosity. For the full length *PICALM and D18-PICALM* isoforms, significant AEI was observed only in the AD40 sample. To evaluate whether a subtle difference in allelic expression may be present, we compared the allelic expression of the *full length PICALM* isoform between the rs3851179 homozygous and heterozygous groups by using a t-test. However, no difference was observed as the average T:G ratio in the rs3851179 homozygous and heterozygous groups was 0.93 ± 0.07 (n = 17) and 0.93 ± 0.06 (n = 18), respectively (Figure 5a, p = 0.81). Likewise, for the *D18-PICALM* isoform, the rs3851179 homozygous and heterozygous groups showed mean T:G ratios of 0.95 ± 0.09 (n = 17) and 1.01 ± 0.12 (n = 18), respectively (Figure 5b, p = value = 0.13). When we evaluated the *D18-19 PICALM* isoform, significant AEI was detected in multiple samples (Figure 5c). We analyzed these results in two ways. First, we compared the frequency of samples with significant AEI between the rs3851179 homozygous and heterozygous individuals by using a Fisher’s exact test; a significant difference between groups was not detected (Figure 5c, p = 0.44). Second, we compared the mean T:G ratio between rs3851179 homozygous and heterozygous individuals by using a t-test. However, the rs3851179 homozygous and heterozygous individuals showed similar values that did not achieve significance, i.e., 1.19 ± 0.16 and 1.11 ± 0.22, respectively (p = 0.08). Hence, rs3851179 heterozygosity was not associated with AEI for these *PICALM* isoforms.

We considered the subset of samples that showed significant AEI further. Among these, AD40 showed significant AEI for each of the isoforms, with the *D18, D18-19* and *full length PICALM* isoforms having allelic T:G ratios of 0.77, 0.53 and 0.77, respectively. As noted above, this finding is consistent with the hypothesis that a rare SNP acts to alter global *PICALM* allelic expression in this individual. Additionally, we noted that the *D18-19 PICALM* isoform showed significant AEI for multiple individuals (Figure 5c). In these eight individuals, the rs76719109T allele was expressed more than the G allele with the ratio ranging from 1.30 to 1.48. This is in contrast to AD40 where the T allele was expressed less than G allele at a ratio of 0.53 (Table 1).

**Table 1:** ***D18-19 PICALM *** **shows significant AEI in nine samples**

**Sample**	***D18-19*** **T:G Counts**	**Ratio (Normalized to genomic ratio)**	**P-Value**
C01	2337:1793	1.30	8.66 x 10^-3^
C11	13415:9508	1.47	7.91 x 10^-34^
C20	745:523	1.43	2.78 x 10^-3^
AD33	6136:4162	1.48	7.06 x 10^-25^
AD43	1743:1321	1.31	9.47 x 10^-3^
AD50	1529:1080	1.41	3.05 x 10^-5^
AD51	5530:4243	1.33	5.27 x 10^-5^
AD54	8893:6548	1.38	2.64 x 10^-14^
AD40	2203:4103	0.53	1.96 x 10^-65^

These *D18-19 PICALM* T:G allelic counts are the summation of three separate runs. AD samples are designated by an AD prefix while non-AD samples are designated with a C prefix.

Since the *D18-19 PICALM* isoform but not overall PICALM showed AEI in multiple samples, we hypothesized that a local SNP influences this splice pattern. To identify this SNP, we sequenced 6400 bp of genomic DNA between exons 17–20 in C11 and AD33, the two individuals showing the highest AEI ratio (Table 1). We identified several SNPs that were heterozygous in these samples including rs588076, rs645299, and rs618629. The MAF for these SNPs in CEU range from 20-31% (Table 2). Additionally, these SNPs are in strong LD with each other (Table 2). Rs588076 and rs618629 reside in intron 17 while rs645299 is within intron 18. We genotyped all samples for rs588076. The frequency of samples showing significant AEI was significantly associated with rs588076 heterozygosity (Figure 5d, p = 9.54 × 10^-5^, Fisher’s exact test). We interpret these results as strongly suggesting that rs588076, or a SNP in strong LD with rs588076, is functional by modulating *PICALM* exon 18–19 splicing.

**Table 2:** Samples with robust AEI are heterozygous for three SNPs

**SNP**	**MAF in CEU**	**LD with rs3851179**	**LD with rs588076**	**LD with rs645299**	**LD with rs618629**
rs588076	0.199	0.336	-	0.539	0.911
rs645299	0.306	0.665	0.539	-	0.525
rs618629	0.242	0.319	0.911	0.525	-

The samples C11 and AD33 were sequenced from exon 17 through exon 20. The samples were heterozygous for the indicated SNPs. These SNPs are in strong LD [23] with each other and have similar frequencies for the minor allele [24].

Since rs588076 is associated with *D18-19 PICALM* AEI and rs3851179 has been robustly associated with AD, we evaluated the extent that rs588076 is associated with AD risk. We evaluated 1789 AD and 2529 non-AD individuals from the Mayo Clinic cohort. Rs588076 was significantly associated with AD risk when analyzed in a logistic regression model (odds ratio = 0.8413, 95% confidence intervals (0.7151-0.9898), p = 0.0372). However, when rs3851179 was added to this logistic regression model, only rs3851179 was significantly associated with AD (Table 3). Haplotypic analysis confirmed that the haplotype containing both rs588076 and rs3851179 report the same association with AD as the haplotype containing only rs3851179 (data not shown). Hence, rs588076 is associated with *D18-19 PICALM* AEI but not AD risk.

**Table 3:** Logistic regression modeling of rs3851179 and/or rs588076 effect(s) on AD

**Model**	**SNP**	**Odds Ratio**	**Confidence Interval**	**P value**
rs588076	rs588076	0.8413	0.7151 - 0.9898	0.0372
rs3851179	rs3851179	0.7786	0.6819 - 0.8891	0.0002
rs3851179 + rs588076	rs3851179	0.7767	0.6604 - 0.9136	0.0023
rs3851179 + rs588076	rs588076	1.005	0.8238 - 1.227	0.959

In addition to the indicated SNPs, these models were adjusted for age of onset, APOE alleles, sex and contributing center.

## Discussion

The primary findings of this report include (i) overall *PICALM* expression shows no evidence of global AEI even when parsed by AD-associated SNPs, (ii) robust global AEI was detected in one sample, suggesting the existence of a rare SNP that strongly modulates *PICALM* expression, and (iii) eight individuals show AEI for the *D18-19 PICALM* isoform that is associated with rs588076. However, rs588076 was not associated with AD risk when considered in a model that also included rs3851179. In summary, analysis of allelic expression has proved a useful tool for the evaluation of cis-acting regulatory polymorphisms and AD risk.

A consistent pattern of AEI in overall *PICALM* expression was not detected. This was unexpected since Xu et al. reported consistent and robust *PICALM* AEI [22]. The reason for different results in these two studies is unclear. The studies are similar in that both used rs76719109 as a reporter SNP and similar although not identical PCR primers. The studies differ in that Xu et al. used an Asian population while this report studied Caucasians. One explanation that would account for the difference in the studies was the presence of a confounding SNP in the Asian population in the genomic primer sequence because much of the AEI in Xu et al. was due to correction for imbalance in gDNA [22], although such a SNP has not yet been reported. We previously reported that the AD-associated SNP rs3851179 was associated with a modest difference in *PICALM* expression when analyzed relative to cell-type specific mRNAs; the minor rs3851179A allele appeared to be expressed modestly higher than the G allele [2]. A similar difference was not observed here. One possible interpretation of these findings is that rs3851179 or its proxy AD SNP acts in a cell-type specific fashion that was discernible in our analysis that included cell-type specific markers. The current AEI study had smaller sample size because only the samples that were heterozygous for rs76719019 or rs592297 were suitable for analysis. However, this would not be expected to affect the AEI results because they rely upon an intra-individual analysis. We interpret these results overall as suggesting that the AD-associated SNP, or its functional proxy, acts in a cell-type specific fashion to modulate *PICALM* expression. This cell-type specific action was not detectable in this AEI study of mRNA derived from multiple cell types.

The second major finding was that robust AEI was detected for all *PICALM* isoforms in one individual, arguing for the existence of a rare functional SNP that strongly modulates total *PICALM* expression. For this individual, the rs76719109G allele was consistently more abundant than the T allele for each *PICALM* isoform. We hypothesize that AD40 is unique among the 42 samples in showing AEI because this sample is heterozygous for a causal SNP. If this causal allele is present in the heterozygous state in 1 of 42 people, this SNP has a minor allele frequency of ~1.2%. Although current sequencing studies of the *PICALM* promoter region have not yet identified candidate functional SNPs for AEI in this sample, these studies are on-going and a SNP that strongly modulates *PICALM* expression would be expected to be a robust AD risk factor.

The third major finding was that the *D18-19 PICALM* isoform showed robust AEI. There was a strong skew towards increased expression of the rs76719109T allele. Sequencing identified several candidate SNPs including rs588076, which is 509 bp downstream of exon 17. This SNP was found to be robustly associated with AEI for *D18-19 PICALM*. There are three possible ways rs588076 could influence *D18-19 PICALM* splicing efficiency: (i) rs588076 is in high LD with a functional SNP that modulates splicing, (ii) rs588076 and other SNPs influence *D18-19 PICALM* splicing in a cooperative manner, and/or (iii) rs588076 directly influences splicing. Further studies are necessary to discern among these possibilities.

The biological significance of the rs588076 association with *D18-19 PICALM* is complex. *D18-19 PICALM* transcripts account for 1-2% of total *PICALM* expression [2]. Thus rs588076 is significantly associated with AEI for a *PICALM* isoform that is relatively rare in brain. Exons 18 and 19 encode a total of 27 amino acids that are part of the carboxyl terminal region required for clathrin binding and endocytosis [25]. Hence, the protein encoded by *D18-19 PICALM* is likely to have reduced function [25]. However, rs588076 was not associated with AD risk and did not enhance the logistic regression model for the rs3851179 association with AD. This leads us to conclude that the rs588076 and *D18-19 PICALM* isoform may be too rare in the brain to influence AD pathogenesis. Interestingly, rs588076 was recently associated with the blood pressure response to ^Ca++^ channel blocking agents [21]. Since rs588076 is associated only with *D18-19 PICALM*, we speculate that this isoform may be more abundant in other tissues and rs588076 actions upon *D18-19 PICALM* mediate this systolic blood pressure phenotype.

## Conclusion

In summary, analysis of allelic expression has shown that compelling *PICALM* AEI was not observed in most brain RNA samples. Strong global AEI was documented in one sample, suggesting the existence of a rare *PICALM* regulatory SNP. A pattern of AEI was clearly discerned for the *D18-19 PICALM* isoform and rs588076 was significantly associated with this pattern*.* Rs588076 was not associated with AD risk although this SNP has been associated with a blood pressure-related phenotype. Allele-dependent expression studies may provide further insights into additional AD-associated polymorphisms.

## Methods

### DNA and RNA extraction from human brain tissue

The RNA and DNA samples for this study were from de-identified AD and non-AD human brain anterior cingulate specimens provided by the University of Kentucky AD Center Neuropathology Core and have been described previously [2,26,27]. The overall dataset included 30 AD samples (14 male, 16 female) and 30 non-AD samples (15 male, 15 female). The age at death for individuals that were cognitively intact, i.e., non-AD, was 82 ± 8 years (mean ± SD, n = 30) while age at death for AD individuals was 82 ± 6 (n = 30). The average post-mortem interval (PMI) for non-AD individuals was 2.8 ± 0.9 hours (mean ± SD, n = 30) while the PMI for AD individuals was similar at 3.4 ± 0.6 hours (n = 30). For the rs76719109 AEI assay, a subset of 35 samples were heterozygous for this SNP and included 18 non-AD (9 male, 9 female) and 17 AD (9 male, 8 female). For the rs592297 AEI assay, a total of 19 out of 60 samples were heterozygous, 13 non-AD (7 male, 6 female) and 6 AD (3 male, 3 female). Preparation of gDNA, RNA and cDNA was performed as described in previous studies [2,26,27]. Although RNA integrity analyses were not performed prior to reverse transcription, others have demonstrated that for qPCR with short amplicons, normalized expression differences are comparable between samples with moderate RNA degradation and those with high integrity RNA [28]. We recognize that the absence of RNA integrity analysis constitutes a caveat of this study.

### Genotyping and sequencing

DNA samples were genotyped for rs3851179, rs76719109, rs592297 and rs588076 by using unlabeled PCR primers and two allele-specific TaqMan FAM and VIC dye-labeled MGB probes (Pre-designed TaqMan SNP Genotyping Assay, Applied Biosystems) on a real-time PCR machine (Chromo4, MJ Research PTC-200).

### Allelic imbalance assay

Rs76719109 is in exon 17. For AEI analysis with rs76719109, *PICALM* was amplified from exon 17 to exon 20 for cDNA and exon 17 to intron 17 for genomic DNA. For rs592297, *PICALM* was amplified from exon 5 to exon 6 for cDNA and exon 5 to intron 5 for genomic DNA. Exon numbering is according to *PICALM*-005 ENST00000393346 for exons 1–16 and exons 17–21 correspond to the final five exons within *PICALM*-002 ENST00000532317 in Ensembl, since no single ENSEMBL transcript includes each of the exons the we identify here [29]. The PCR primers included Ion Torrent adapters, individual barcodes and DNA sequence flanking the region of interest (Table 4). Each PCR reaction (50uL) contained 1x PCR buffer, 20 ng cDNA or 100 ng gDNA, 1uM of forward and reverse primer, 0.1 mM dNTP and 0.5 Units Taq (Platinium Taq, Invitrogen). Cycles consisted of pre-incubation at 95°C for 2 minutes, followed by 28 cycles of 95°C for 15 s, 60°C for 30s and 72°C for 60s followed by incubation at 75°C for 7 minutes. To acquire equal representation from each sample, relative amounts of PCR product were quantified by subjecting 10 uL of PCR product to electrophoresis on 7.5% polyacrylamide gels and SYBR gold staining relative to a Low DNA Mass Ladder (Invitrogen). Approximately 2 ng of each individual’s cDNA and gDNA PCR products were pooled, purified using Agencourt AMPure XP and subjected to Ion Torrent sequencing on a Ion Torrent 316 chip (Ion PGM Sequencer).

**Table 4:** PCR primers for rs76719109 and rs592297 AEI assay

**Assay**	**DNA**	**Primer Sense**	**Primer Sequence**
rs76719109	cDNA	Sense	5′CCATCTCATCCCTGCGTGTCTCCGACTCAGxxxxxTGGAGTCAACCAGGTGAAAA
	cDNA	Anti-sense	5′ CCTCTCTATGGGCAGTCGGTGATTTGGTTGCGTCATTACAGGA
	gDNA	Sense	5′CCATCTCATCCCTGCGTGTCTCCGACTCAGxxxxxTGGAGTCAACCAGGTGAAAA
	gDNA	Anti-sense	5′ CCTCTCTATGGGCAGTCGGTGATAGGAGCTTTTTCAACTCACCA
rs592297	cDNA	Sense	5′CCATCTCATCCCTGCGTGTCTCCGACTCAGxxxxxTGAACACAGAAAAACTCCTAAAAA
	cDNA	Anti-sense	5′ CCTCTCTATGGGCAGTCGGTGATGGCAGCATTTATTACCCCATT
	gDNA	Sense	5′CCATCTCATCCCTGCGTGTCTCCGACTCAGxxxxxTGAACACAGAAAAACTCCTAAAAA
	gDNA	Anti-sense	5′ CCTCTCTATGGGCAGTCGGTGATTCTGTGAAAACTTGAGGTTAAAAA

"xxxxx" denotes 5 nucleic acid barcode, which is unique for each individual. Note that gDNA and cDNA were amplified by the same barcoded forward primers. Genomic DNA and cDNA were differentiated by the reverse primers.

### Data extraction and analysis of allelic mRNA expression

Allelic counts were extracted from DNA sequences by using Perlscript in a three-step fashion: (i) sequences corresponding to each sample were separated based on their barcode, (ii) gDNA and cDNA were then separated based on the presence of intronic and exonic sequences, respectively, and (iii) allele counts were obtained by using sequences that bridged the SNP of interest.

### Standard curve generation

One rs76719109 homozygous major (GG) and one homozygous minor (TT) individual was selected based on similar qPCR copy numbers. Five dilutions were prepared with different ratios of each individual’s cDNA: 1:4, 1:2, 1:1, 2:1, and 4:1. These samples were PCR amplified and subjected to sequencing as described above.

### Statistical analysis

Analysis of allelic counts was based upon the assumption that transcript read counts follow a Poisson distribution [30]. As such, each allele from the heterozygous SNP was used to define two random variables. Following the rs76719109 example of a G/T SNP, we denote the pair of transcript counts as *G* ~ *Poisson* (*λ*_*G*_) and *T* ~ *Poisson* (*λ*_*T*_). That is, G and T are Poisson-distributed random variables with means *λ*_*G*_ and *λ*_*T*_, respectively. It can then be readily shown that for a given pair of realized transcripts counts, G = g and T = t, the transcript count of either allele is binomially distributed with success probability equal to a ratio of component means. That is, $G\;|\;G+T=g+t\;\sim \;\text{Bin}\;\left(g+t,\;p=\frac{\lambda_{G}}{\lambda_{G}+\lambda_{T}}\right)$. Testing for AEI then simplifies to an examination of the null hypothesis that the pair of transcript counts comes from the same distribution, i.e., that *λ*_*G*_ = *λ*_*T*_ which is equivalent to testing $H_{0}:\;p=\frac{1}{2}\;\left[G\;|\;G+T=g+t\;\sim \;\text{Bin}\;\left(g+t,\;\frac{1}{2}\right)\right]$. This null hypothesis agrees with the intuition that when the total of transcript counts is known, the number generated from a specific allele is essentially a sequence of independent, equally probable trials. Thus, rejection of this null hypothesis corresponds to AEI.

Measuring transcripts from genomic DNA is one way of correcting for the possibility of differential experimental error between allele transcript counts. Conceptually, one could adapt methods for determining AEI by an appropriate adjustment with the ratio of reads from gDNA as these reads should theoretically come from the same distribution regardless of AEI (Fardo et al., unpublished). Alternatively, it can be assumed that one allele is derived from a distribution with an inflated mean solely due to experimental error (i.e., under the null hypothesis of no AEI). In this case, we have that the means of the transcript reads satisfy either *λ*_*G*_ = (1 + *δ*)*λ*_*T*_ or (1 + *δ*)*λ*_*G*_ = *λ*_*T*_. Here, the probability parameter, p, for the count probability in the AEI test becomes $\frac{1+\delta }{2+\delta }$ or $\frac{1}{2+\delta }$, respectively. For our gDNA data, we have a maximum 8.5% increase of one allele over the other and chose to conservatively assume a 20% mean increase (i.e., *δ* = 0.2). We then calculate the AEI test p-value from the lesser-significant test of $H_{0}:\;p=\frac{1+\delta }{2+\delta }$ and $p=\frac{1}{2+\delta }$.

### Genotype association with AD risk

The Mayo Clinic dataset has been described previously [31,32]. Briefly, the Mayo Clinic dataset contained 1789 cases and 2529 non-ADs collected from six centers from the US and Europe as described [32]. Direct genotyping of rs3851179 and rs588076 was performed using a TaqMan SNP genotyping assay in an ABI PRISM 7900HT Sequence Detection System with 384-well block module from Applied Biosystems (California, USA). First-pass genotype cluster calling was analyzed using the SDS software version 2.2.3 (Applied Biosystems, California, USA). Variants passed Hardy-Weinburg (P > 0.05) and minor allele frequencies are consistent with public databases (EVS, HapMap, 1000G). Association testing for rs3851179, with and without rs588076, was carried out in PLINK [33] by using an additive logistic regression model corrected for appropriate covariates; diagnosis age, *APOE* 4, *APOE* 2, sex and contributing center.

## Competing interests

The authors declare that they have no competing interests.

## Authors’ contributions

IP carried out the molecular genetic AEI studies and analysis and helped to draft the manuscript. CM and SG evaluated the SNP’s association with AD risk. DWF participated in the design of the study and performed the statistical analysis. SE helped design the study and draft the manuscript. All authors read and approved the final manuscript.

## Supplementary Material

Additional file 1: Table S1 *PICALM* AEI analysis of AD40 shows significant unequal rs76719109T to G allele ratios. Click here for file
