# Supplementary material for: An intronic PICALM polymorphism, rs588076, is associated with allelic expression of a PICALM isoform
Source: Mol Neurodegener. 2014 Aug 29;9:32. doi: 10.1186/1750-1326-9-32 (PMC4150683; doi:10.1186/1750-1326-9-32)
Supplement: Additional file 1: Table S1 — PICALM AEI analysis of AD40 shows significant unequal rs76719109T to G allele ratios. [file 1750-1326-9-32-S1.doc]

**Supplemental Table 1. *PICALM* AEI analysis of AD40 shows significant unequal rs76719109T to G allele ratios.**

| **Rs76719109** | Counts | Ratio (T/G) | mRNA ratio normalized to genomic ratio | P-value |
| --- | --- | --- | --- | --- |
| Genomic **G** Allele | 40357 | 1.01 |  | 0.89 |
| Genomic **T** Allele | 40880 |  |  |  |
| mRNA **G** Allele | 88400 | 0.77 | 0.76 | 6.76 x 10^−58^ |
| mRNA **T** Allele | 68141 |  |  |  |
| ***Isoforms*** |  |  |  |  |
| D18 **G** Allele | 17777 | 0.78 | 0.77 | 3.63 x 10^−09^ |
| D18 **T** Allele | 13909 |  |  |  |
| *D18-19* **G** Allele | 4103 | 0.54 | 0.53 | 1.96 x 10^−65^ |
| *D18-19* **T** Allele | 2203 |  |  |  |
| Full length **G** Allele | 66520 | 0.78 | 0.77 | 1.88 x 10^−30^ |
| Full length **T** Allele | 52029 |  |  |  |

Genomic DNA analysis shows an overall equal allelic representation while mRNA analysis shows significant AEI. This AEI was present in each of the three isoforms.
